# Seeing nanoscale electrocatalytic reactions at individual MoS_2_ particles under an optical microscope: probing sub-mM oxygen reduction reaction†

**DOI:** 10.1039/d4fd00132j

**Published:** 2024-07-10

**Authors:** Nikan Afsahi, Zhu Zhang, Sanli Faez, Jean-Marc Noël, Manas Ranjan Panda, Mainak Majumder, Naimeh Naseri, Jean-François Lemineur, Frédéric Kanoufi

**Affiliations:** a Université Paris Cité, CNRS, ITODYS F-75013 Paris France frederic.kanoufi@u-paris.fr jean-francois.lemineur@u-paris.fr; b Nanophotonics, Debye Institute for Nanomaterials Science, Utrecht University 3584 CC Utrecht The Netherlands; c Nanoscale Science and Engineering Laboratory (NSEL), Department of Mechanical and Aerospace Engineering, Monash University Clayton VIC 3800 Australia; d ARC Research Hub for Advanced Manufacturing with 2D Materials (AM2D), Monash University Clayton VIC 3800 Australia; e Department of Physics, Sharif University of Technology Tehran 11365-9161 Iran

## Abstract

MoS_2_ is a promising electrocatalytic material for replacing noble metals. Nanoelectrochemistry studies, such as using nanoelectrochemical cell confinement, have particularly helped in demonstrating the preferential electrocatalytic activity of MoS_2_ edges. These findings have been accompanied by considerable research efforts to synthesize edge-abundant nanomaterials. However, to fully apprehend their electrocatalytic performance, at the single particle level, new instrumental developments are also needed. Here, we feature a highly sensitive refractive index based optical microscopy technique, namely interferometric scattering microscopy (iSCAT), for monitoring local electrochemistry at single MoS_2_ petal-like sub-microparticles. This work focuses on the oxygen reduction reaction (ORR), which operates at low current densities and thus requires high-sensitivity imaging techniques. By employing a precipitation reaction to reveal the ORR activity and utilizing the high spatial resolution and contrast of iSCAT, we achieve the sensitivity required to evaluate the ORR activity at single MoS_2_ particles.

## Abstract 2

Interferometric scattering microscopy (iSCAT) allows visualizing individual petal-like MoS_2_ sub-microparticles and monitoring *operando* their activity towards oxygen reduction reaction.

## Graphical abstract

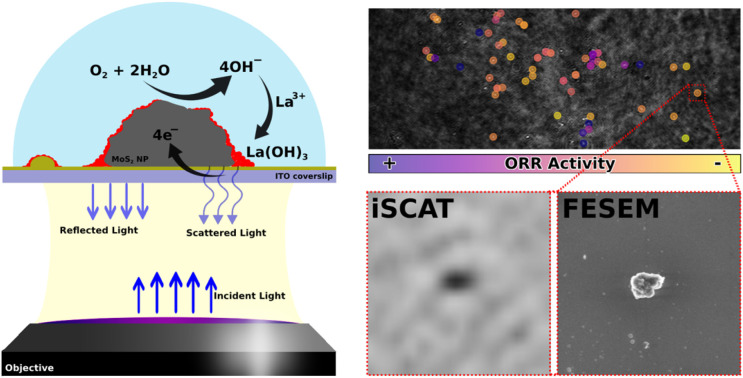

## Introduction

Understanding the oxygen reduction reaction (ORR) is relevant in many chemical sectors, ranging from the activation of O_2_ for enabling chemicals oxygenation under mild conditions,^1,2^ the depollution of water contaminants by electro-Fenton processes,^3^ to energy production or storage with the search for earth-abundant catalysts for fuel cells^4^ or the development of viable strategies for metal–air, *e.g.* Li–air, batteries.^5,6^

ORR is a complex process involving up to 4e^−^ + 4H^+^ (or 4 Li^+^ in battery) transfers, depending on the nature of the electrode or catalyst used, or applications sought. Mechanistic understanding of the ORR is crucial to control the reaction selectivity. Particularly, it is important to control or avoid the formation of radical intermediates, known as reactive oxygen species, ROS. Partially reduced ROS can indeed be produced at different extents depending on the nature of the electrocatalysis (heterogeneous *vs.* homogeneous: surface *vs.* molecule) employed to perform the ORR.^7–9^ Considerable understanding of the heterogeneous ORR mechanisms is achieved from the study of model single crystal electrodes, by inspecting how ORR intermediates are revealed by adsorption features in voltammograms.^8,10–12^ The involvement of such intermediates, at ideal and non-ideal electrocatalysts, is confirmed by *in situ* (or *operando*) complementary characterizations.^13^ It can be afforded by electrochemical approaches in generation-collection mode, using rotating ring-disk electrodes, or local electrochemical probing by scanning electrochemical microscopy (SECM),^2,7^ in-flow detection of electrode dissolution processes,^13^ or complementary spectroscopic inspections, such as Raman or IR.^9,14^

Most of these studies highlight the importance of controlling the electrode surface, particularly by introducing nanostructuration, by engineering facets orientations and surface strain, in order to improve the sluggish kinetics of the ORR.^4,10,12^ While this supports the use of nanoparticles or nanostructured electrodes as electrocatalysts for ORR in real electrochemical devices, evidencing or imaging the extent of ORR at the nanoscale is quite challenging. Pure electrochemical-based strategies rely on probing locally the dissolved ORR intermediates using microelectrodes in the SECM configuration,^2,7,15^ or using nanoparticles-based nanoelectrodes,^16^ or imaging the local ORR activity at Cu or metal–organic frameworks using nanopipettes confining nanoelectrochemical cells.^17,18^ Complementary imaging instruments have also been used to reveal the extent of ORR at nanostructured objects. AFM allowed imaging the dissolution of Pt micro/nanoelectrodes during ORR,^19^ while single molecule fluorescence microscopy could reveal the 2e^−^ reduction of O_2_ (production of H_2_O_2_) at single Fe_3_O_4_ nanoparticles (NPs).^20^ Finally, the ultimate nanoscale identification of ORR catalytic surface sites was enabled by noise fluctuation monitoring in electrochemical scanning tunneling microscopy (n-EC-STM).^21–23^

**Fig. 1:**
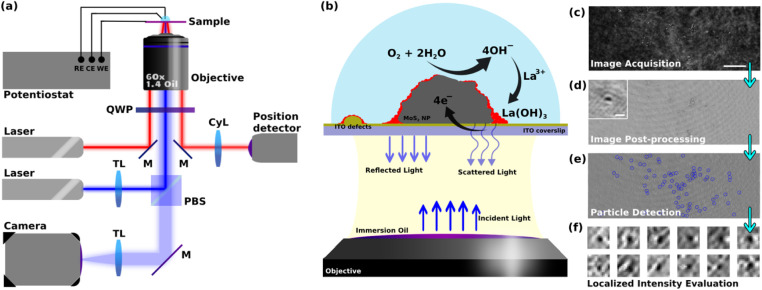
(a) Schematic illustration of the iSCAT microscope used including the electrochemical workstation, imaging optics, position detection optics, objective, and camera (the abbreviations stand for M: mirror, TL: tube lens, CyL: cylindrical lens, QWP: quarter-wave plate, PBS: polarizing beam splitter, RE: reference electrode, WE: working electrode, CE: counter electrode). (b) Schematic illustration of electrochemical cell area, ORR pathway reactions, and optical elements involved in construction of iSCAT images. (c) Typical raw image obtained from iSCAT setup (scale bar 5 μm). (d) Typical post-processed interferometric image (inset: zoomed-in image showing the typical detected interference patterns, scale bar 200 nm). (e) Typical result for the detection of local metal hydroxide deposition. (f) Typical zoomed-in images of particles used in studying the local intensity changes.

Optical microscopy has also been used to image a variety of electrochemical processes,^24–26^ while more rarely to image the ORR at the nanoscale.^20^ Herein we pursue our former methodology coupling refractive-index based optical microscopy and electrochemistry to image reactions at individual NPs.^25–30^ The microscope imaging instrument and methodology are presented together in Fig. 1 and detailed below. It is based on an interferometric imaging mode enabling an increased sensitivity in the detection of local changes in refractive index near an electroactive domain. In order to detect and image the ORR at individual active sites, one needs to transform this reaction into an optical readout that can be detected at high throughput (in space and time). While fluorescent labeling, *e.g.* with resorufin, has been employed to reveal the production of H_2_O_2_ during ORR in a 2e–2H^+^ transfer process,^20^ here we rather propose a refractive-index labeling to reveal the ORR. The methodology, summarized in Fig. 1, uses the precipitation of a Lewis-acid metallic cation into a metal hydroxide^30^ (herein La^3+^ and La(OH)_3_, respectively) during the production of HO^−^ associated to the ORR reaction. Henceforth, active sites of ORR will be footprinted optically as regions where local deposition of La(OH)_3_ is detected. In order to increase the sensitivity of the detection of the active sites of ORR, we used the laser-illuminated interferometric scattering microscopy, also known as iSCAT, which has shown single biomolecule sensitivity.^31,32^ Despite its exceptional ability to optically probe mass changes at surfaces, the iSCAT microscopy has rarely been used for probing electrochemical reactions. The iSCAT microscope has evidenced double layer charging at the micro- to nanoscale,^33,34^ or has imaged the dynamics of Li-ion insertion into active microparticles for batteries.^35–37^

Herein, we illustrate the potential of iSCAT to image electrocatalytic reactions using the electrochemical ORR at MoS_2_ particles deposited onto an indium tin oxide, ITO, electrode. This work particularly presents the methodology enabling to image optically the ORR reaction. After presenting the methodology, based on a refractive-index labeling of the reaction (more precisely of the formation of HO^−^), we first show how the strategy can be used to reveal the ORR at the current collector, an optically transparent ITO electrode. Indeed the low electrocatalytic activity of the current collector electrode is often a requisite to determine the electrocatalytic activity of individual particles, for example in nanoimpact electrochemistry for which active collectors can be used at the expense of more complex data interpretation.^38,39^ Next the strategy is employed to image the ORR at individual sub-micrometric MoS_2_ particles. There has been considerable recent efforts in imaging by local electrochemical probes, mostly AFM-SECM or nanopipettes, the local electrocatalytic activity of MoS_2_ 2D-layered materials.^40–44^ For hydrogen evolution reaction (HER), a preferential, up to 2 orders of magnitude higher, edge-activity has been demonstrated by nanopipette electrochemistry. However much less was shown for the ORR, in part since it operates at lower current densities than HER. Meanwhile, the promising edge-activity of MoS_2_ has fueled interest in synthesizing edge-abundant MoS_2_, such as fractal or porous nanostructures,^45–47^ for which optical microscopy may play a pivotal role for imaging local electrocatalytic activity. It is illustrated here for probing the electrocatalytic ORR activity at single petal-like MoS_2_ particles.

## Results and discussions

### Methodology

This work uses 2H-MoS_2_ flower or petal-like nano- to microparticles which are drop-cast onto an optically transparent electrode, in order to optically monitor, *operando*, the electrochemistry of ORR at the single particle level. The size distribution of the particles was studied by detecting the particles in multiple SEM images and using the Canny edge detection algorithm. The size of the particles was defined as the diameter of the biggest circle encircling the detected particles (ESI, Fig. S1†). This calculation shows that the particles’ size ranges from 200 nm up to 5 microns. The size distribution shows a peak at around 400 nm ± 100 nm and more than 80% of the particles fall into a range of sizes less than 1.2 microns (Fig. S2 in ESI†). A transparent ITO coated glass coverslip electrode was chosen as the current collector to study the electrochemistry of the particles. The opto-electrochemical measurements were conducted using a modified electrochemical cell comprised of either the bare ITO electrode or covered with MoS_2_ particles used as a working electrode, a Ag/AgCl wire as the reference electrode, and a 250 μm diameter Pt wire as the counter electrode. The optical analyses were performed using a custom-built interferometric scattering (iSCAT) apparatus (Fig. 1a), which is an inverted microscope utilizing a 450 nm wide-field incident laser beam as the primary illumination source. An oil-immersed 60× objective was used to illuminate, from the backside, the interface of the WE formed with the electrolytic solution. It also collects the back-scattered light from the particles present on the WE surface as well as the reflected light from the electrochemical interface, as schematized in Fig. 1b. The iSCAT image was then formed from the interference between the back-scattered light and the reflected beam which were transmitted through the objective to a s-CMOS camera. Fig. 1c shows such a raw iSCAT image. It is made of interference fringes, whether constructive or destructive, depending on the local structure of the interface. Particles, such as defects on the ITO layer or MoS_2_ particles lying on the electrode surface, are also detected, as evidenced from the zoomed-in image of a MoS_2_ particle in the inset of Fig. S4.† This interferometric mode of detection is highly sensitive to subtle changes in local dielectric composition and refractive index, allowing for the tracking of slight alterations in the arrangement and characteristics of the particles and the surface.

The method employed to probe ORR reaction (eqn (1)) leverages the idea that local ORR at active sites is associated to local changes in pH around these active sites by local production of hydroxide ions (OH^−^). The ORR reaction in eqn (1) is considered to be a 4e–4H^+^ transfer process, producing 4 HO^−^ in a non-acidic medium. The path that considers the formation of H_2_O_2_ by a 2e–2H^+^ transfer would equivalently produce 2 HO^−^ (half as much as by eqn (1)), it would then be probed in the same way as the 4e–4H^+^ transfer process, but with a HO^−^ production rate twice as low. This work, describing the overall monitoring methodology, does not pretend to deal with the possibility of differentiating the 2 *vs.* 4 electron paths of ORR. In the presence of Lewis acid metallic cation, here 1 mM La^3+^, the ORR can lead to the precipitation of lanthanum hydroxide (La(OH)_3_) (eqn (2)). These precipitations are then expected to alter the local optical properties of the interface near the active sites, modifying the interference patterns observed in the iSCAT images. This idea and the aforementioned methodology are encapsulated in the schematic presented in Fig. 1b.

1 (in non-acidic medium)  O_2_ + 2H_2_O + 4e^−^ → 4OH^−^

2 La^3+^ + 3OH^−^ → La(OH)_3_

The images of the region of interest (ROI) acquired during the electrochemical reaction (Fig. 1c) were next post-processed using python-based automated routines to extract the relevant information related to the ORR from the raw data (Fig. 1d). This procedure mainly consists of correcting for the spatial drift in the video, performing background subtraction, and synchronizing the optical data with the electrochemical data. The ESI Video S1† gives an example of a post-processed video recorded during a cyclic voltammetry experiment performed at an MoS_2_ drop-cast ITO electrode in contact with a pH = 5, 0.1 M KCl + 1 mM La^3+^ solution. While most of the particles and ITO heterogeneities can be readily distinguished on the raw images (Fig. 1c), image post-treatment (Fig. 1d) then clearly reveals slight changes in iSCAT contrast in the vicinities of these particles (Fig. 1e and f, discussed below). This allowed for (1) identifying and labeling electroactive sites (*i.e.* MoS_2_ particles or ITO surface defects), (2) patching the adjacent environment around these sites (Fig. 1f), and (3) analyzing the optical intensity dynamics in these patches which is then used to study the local dynamics of the electrochemical process in these identified electroactive regions. These optical analyses were then related to the macro-scale electrochemical results.

### Electrochemical and ensemble-averaged optical responses

**Fig. 2:**
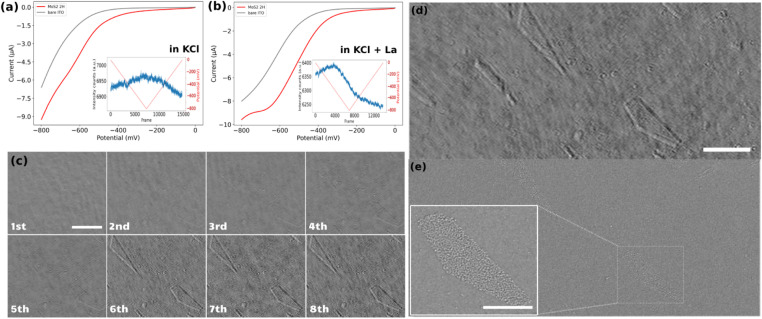
Forward scan of the cyclic voltammetry for the ORR at MoS_2_-coated (red line) and bare (grey line) ITO samples in (a) 0.1 M KCl solution pH = 5 or (b) solution containing 0.1 M KCl and 1 mM La^3+^ cations at pH = 5. Insets show the electrode potential function (red line) and iSCAT optical intensity changes averaged over the whole imaged region (blue line) for MoS_2_-coated electrodes. (c) The iSCAT images of 8 successive cycles between 0 and −0.8 V of cyclic voltammetry for the bare ITO sample (scale bar = 5 μm). Same region of interest of the ITO surface imaged by (d) *operando* iSCAT image (scale bar 5 μm), (e) post-mortem *ex situ* SEM image (inset shows the magnified image of ITO’s surface defects (scale bar = 2 μm)).

The electrochemical response of the samples was examined using cyclic voltammetry (CV). For clarity, only the forward scans of the CVs are presented in Fig. 2a and b. These curves represent the potential–current profiles for bare ITO (indicated by the grey line) and ITO decorated with drop-cast MoS_2_ particles (represented by the red line), in a pH = 5, 0.1 M KCl solution in the absence (Fig. 2a) and in the presence (Fig. 2b) of 1 mM La^3+^ cation. The results indicate that the overall electrochemical behavior of the two samples remains consistent across the two different electrolytes, despite a slight shift in the onset potential for the cathodic current. It might be due to different factors, such as different particle densities on both substrates, or the presence of La^3+^ cations which buffers the pH of the electrolyte close to its initial value, preventing it from becoming more alkaline during the ORR. This stabilization of pH can enhance the electrochemical response of the sample in the La-containing KCl solution compared to the pure KCl solution.

These electrochemical experiments are monitored optically by recording iSCAT raw images of a 53 × 20 μm^2^ region of the electrodes at a rate of 90 frames per second. The inset graphs in Fig. 2a and b display the optical intensity fluctuations, averaged over the fully imaged region, for the MoS_2_ sample in the two different electrolytes. The curve corresponding to the sample in the KCl electrolyte shows a triangular rise and fall in average optical intensity over the whole cycle of a cyclic voltammetry experiment. Specifically, there is an almost linear increase in average intensity during the cathodic forward scan and a linear decrease during the anodic backward scan of the CV. These linear variations in average intensity can be attributed to changes in the local refractive index due to charge accumulation on the ITO surface during the double layer charging and discharging. It supports previous demonstration of the potentiality of refractive-based microscopies for imaging double layer electrochemical charging at nanoparticles.^33,48–50^

Conversely, the situation is different when La^3+^ ions are present in the electrolyte. The post-treated videos corresponding to the CV on the ITO electrode in the presence of La^3+^ ions is provided as ESI Video S2.† The average optical intensity curve for the sample in the electrolyte containing La^3+^ ions exhibits an initial increase in average intensity (double layer charging) followed by a strong decrease in average intensity starting before the reversal potential of the CV and continuing over the backward scan. This is then succeeded by a linear decrease in intensity corresponding to the double-layer discharging. The abrupt decrease in intensity can be attributed to a change in the refractive index caused by the precipitation of lanthanum hydroxide in regions containing ORR electrocatalytically active sites on the electrode.

### Bare ITO electrochemical performance towards ORR

Before analyzing the electrocatalytic activity towards ORR of individual MoS_2_ particles we first assess the electrochemical performance of the bare ITO electrode. This was studied through 8 successive voltammetric cycles (1st cycle is viewed in Video S2†). Fig. 2c displays the iSCAT background-subtracted frames captured at the end of each successive cycle. Comparing the images of the ITO electrode at the end of the 1st and 8th cycles shows that at least two geometric features (a triangle region in the upper left corner and a pentagon at the lower right corner of the images) appeared during the successive ORR cycles. This sequence illustrates that these new features appear progressively, as soon as the 2nd cycle, but with most significant changes in local intensities during the 6th cycle. These observations suggest that while the bare ITO surface exhibits some performance toward the ORR, significant activity is localized in specific regions, identified by the contrasted areas in the iSCAT images. Moreover, it can be suggested that some sort of activation process is required for the ITO surface to exhibit marked electrochemical activity since much less La(OH)_3_ precipitation occurs during the first cycles.

To confirm the localized ITO surface activity, the exact same region observed *operando* by iSCAT (frame in Fig. 2d) was imaged post-mortem using field emission scanning electron microscopy (FESEM) (Fig. 2e). The FESEM image indicates that the active regions detected in the iSCAT image correspond to defects on the ITO surface, which are typically of several μm^2^ and distributed over the entire ITO coverslip. This particularly confirms previous electrochemical imaging studies that revealed the heterogeneous local electrochemical activity of ITO surfaces.^51–53^ The inset in Fig. 2e provides a magnified view of these defects. These results suggest that the most electrochemically active regions on the ITO surface are located at the edges of these surface defects. Furthermore, these images demonstrate the method’s sensitivity in detecting active regions on conducting surface.

### iSCAT analysis of ORR at MoS_2_ nanoparticles

**Fig. 3:**
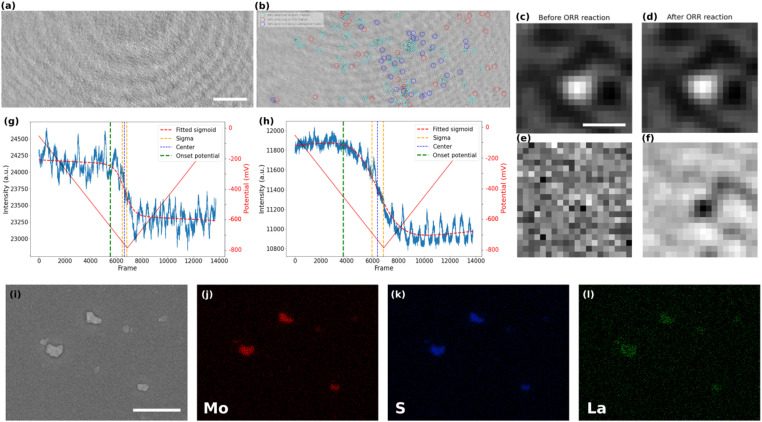
iSCAT image of (a) bare ITO sample (scale bar = 5 μm) and (b) MoS_2_ nanoparticles drop-cast on the ITO coverslip. Magnified image of a MoS_2_ particle (scale bar = 500 nm) (c) before the ORR and (d) after the ORR. Magnified background-subtracted iSCAT image of MoS_2_ particle (e) before the ORR and (f) after the ORR. Intensity *vs.* video frame number (lower axis) and potential (upper axis) of (g) bare ITO and (h) MoS_2_ nanoparticles drop-cast on the ITO. (i) SEM image of MoS_2_ nanoparticles on the ITO coverslip (scale bar = 1 μm). Elemental EDX mapping of (j) Mo, (k) S, and (l) La.

The local activity of the ITO electrode towards ORR could only be detected after several repeated cycles. It can therefore be considered a rather inert current collector for ORR, and may allow the study of particles active towards ORR. The post-treated video corresponding to the CV of the MoS_2_-coated electrode in the presence of La^3+^ is shown in Video S1.† The iSCAT images of bare ITO and MoS_2_ coated sample, prior to the beginning of the electrochemistry of ORR, are presented in Fig. 3a and b, respectively. The difference between the two images can be seen more clearly in the magnified images of local regions (Fig. S3 in the ESI†). The particles on the substrate were detected, labeled, and studied using an automated Python routine. The particles’ detection was achieved in two steps.

First, the nanoparticles were located in the first frame of the video and their position, optical intensity and apparent optical size were recorded. The size distribution of particles that are detected in the first image is computed by fitting a Gaussian function to the magnified image of each particle. The apparent optical size of the particle was then defined as the Full Width at Half Maximum (FWHM) of this fitting function. Such fit is presented in Fig. S4 in the ESI,† while an example of the zoomed-in raw iSCAT image of a MoS_2_ particle is provided in Fig. 3c. The histograms of apparent optical particle size distributions (Fig. S5 in the ESI†) show that the size of the detected particles are all above 350 nm, which is larger than the diffraction limit according to the microscope used (*λ*/2NA ∼ 160 nm with NA = 1.4 the numerical aperture of the microscope objective and *λ* = 450 nm the illumination wavelength). The population of particles optically imaged fits within the distribution of particle size evaluated by FESEM (Fig. S2, ESI†) except for the smallest (<300 nm) and largest (>1.2 μm) which are not represented. However, the integrated optical intensity increases with the apparent optical size (Fig. S6, ESI†), it is likely that the smallest particles are screened by the largest one and are not directly detected optically, unless using a higher illumination power.

In a second step, the image sequence recorded during the CV experiment was analyzed. Fig. 3d represents the zoomed-in raw iSCAT image of the same particle in Fig. 3c but after the ORR reaction has taken place. For better visualization of the ORR activity at the individual particle level, the optical images were (1) filtered by a moving average filter to reduce the noise and (2) the first frame of the filtered image sequence, which can be regarded as the static background for all later frames, was systematically subtracted from the other images. The result of the image processing is illustrated in Fig. 3e and f which show the zoomed-in images in a particle region (same region as in Fig. 3c and d) before and after the ORR reaction, respectively. The background subtraction allows to reveal only the changes in intensity during the electrochemical process that cannot be seen by the naked eye from raw images. As a result, a dark feature appears upon ORR in Fig. 3f while nothing is observed before the reaction. This darkening is consistent with the decrease in intensity observed on average over the whole imaged surface as presented in Fig. 2b.

This image processing also revealed active sites towards ORR in regions where particles were not optically detected in the first frame (before potential application). One could postulate that some particles are too small to be detected on the first image in the observation conditions and without background subtraction, meaning in the sub-400 nm range based on the optical size distribution presented in Fig. S5.† Noteworthy, those particles may have been detected using higher light power, but the largest ones would saturate the detector and one would not be able to image their ORR activity.

Interestingly, the image processing also allowed identifying particles which might be electrochemically inactive, since some of them were detected in the first frame but did not show intensity fluctuation (no metal hydroxide precipitation and therefore no local ORR activity). A reason for such low activity might be due to a poor electrical contact between the particle and the ITO substrate.^54^

A color code has been defined to summarize the different events observed at the electrode surface and is presented in Fig. 3b. The particles detected only in the first frame are marked in red (inactive) while the particles detected only in the background-subtracted image after ORR are represented by blue circles (smaller particles and active) and the particles that are detected with both approaches are shown in cyan (larger particles and active). This figure shows that the method can be used to distinguish between active and inactive particles or different families of particles. Moreover, it shows the high sensitivity of the method in detecting electrocatalytically active sub-micrometer regions even when they are initially not visible optically (blue population).

The precipitation of La(OH)_3_ around and onto the active particles and on the ITO surface is also confirmed by post-mortem energy-dispersive X-ray spectroscopy (EDX) analysis. The elemental mappings in Fig. 3j–l show the spatial distribution of Mo, S, and La for the region of interest presented in the SEM image of Fig. 3i. One can clearly notice the selective deposition of La around the MoS_2_ particles after the ORR process and still present after removing the electrolyte solution from the sample’s surface. It supports the La(OH)_3_ precipitation associated to the electrochemical activation of the electrode surface. The higher deposit onto the MoS_2_ particles than on the ITO surface suggests higher ORR activity of the MoS_2_ than for the ITO, which is also supported by the lower overpotential for ORR on the MoS_2_ covered electrode (Fig. 2b).

The electrochemical experiment being monitored in real-time, we can further infer the dynamics of the ORR at the individual particle level. For that purpose, the variations in the intensity integrated in a 15 px × 15 px (or ∼1 μm × 1 μm) region around each detected particle in the MoS_2_ sample and in different regions on the bare ITO sample were carefully studied. Examples of the intensity profiles of a given ITO region and of the MoS_2_ particle shown in Fig. 3c, are given respectively in Fig. 3g and h. The periodic (*ca.* 7 Hz) fluctuations observed in these profiles are related to a periodic motion of the large interference circular fringes, most likely due to vibrations of the setup. For all the active regions discussed and categorized above, intensity profiles were plotted similarly to the plots presented in Fig. 3g and h. These plots represent the dynamics of the precipitation of La(OH)_3_ on these different regions and therefore allow inferring the dynamics of the ORR activity at the single particle level. The intensity profiles were then fitted using a sigmoidal function (eqn (3)) to extract the onset potential where the light intensity deviates from the background level, revealing the onset potential for the La(OH)_3_ precipitation on and around the particles.

3 *I* = *I*_bkgd_ − *I*_ORR_/(1 + exp(−*α*(*t* − *t*_1/2_)))

where *I* is the measured local intensity, *I*_bkgd_ is the local background intensity, *t* is the time and *I*_ORR_ corresponds to the amplitude of the sigmoid which characterizes the optical mass of deposited La(OH)_3_. Therefore, the higher *I*_ORR_, the higher the mass and the more effective is the ORR.

From the half-wave time, or center, *t*_1/2_, and the *α* parameter, characterizing the slope of the sigmoid, one can evaluate the onset time and the onset potential of the precipitation and therefore of the ORR activation for each active domain. The value of *α* then shows the steepness of the fitted function or how fast the function makes a transition from its upper to its lower asymptote. Based on the value of this variable and the root mean square of the noise of the optical signal, one can define the onset time of deposit detection, at which the intensity curve starts to fall below the noise level due to contrast darkening triggered by La(OH)_3_ precipitation.

### iSCAT and the kinetics of ORR

**Fig. 4:**
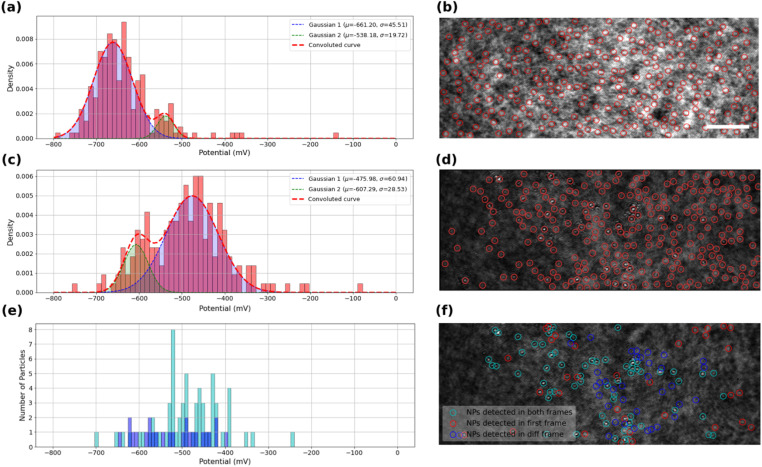
Calculated onset potential probability histogram and corresponding fitted Gaussians for automatically detected scattering regions (a) on bare ITO coverslip, (c and e) on MoS_2_ drop-cast ITO coverslip, using (c) high threshold (maximum number of detected features) or (e) low threshold, detection. Corresponding first frames of the recorded videos with selected regions for intensity analyses for (b) bare ITO coverslip (scale bar 5 μm), (d and f) MoS_2_ drop-cast ITO coverslip. In (e and f) particles are classified into three categories: those detected in the first raw image and active during ORR (cyan), those active but not detected on the first image thresholding (blue) and detected on the first image and inactive (red). The distribution in (e) shows the onset potentials of detected and active particles (cyan) and not detected but active particles (blue).

The kinetics of the oxygen reduction reaction (ORR) on the samples were analyzed using the data extracted from intensity profiles. The values onset potentials are plotted in Fig. 4a for bare ITO surface, and in Fig. 4c and e for MoS_2_-coated ITO electrode, with each onset potential distribution peak fitted using a Gaussian function.

Fig. 4a displays the extracted onset potentials for the bare ITO sample across the entire potential range. These onset potentials were extracted by analyzing the intensity profiles at all light scattering regions of the ITO electrode in the iSCAT image shown in Fig. 4b. These regions of interest, ROIs, on bare ITO are revealed by a stronger illumination of the bare ITO sample compared to the MoS_2_-coated one as suggested by the twice higher optical intensity and 5-fold higher noise fluctuations observed comparing Fig. 3g and h. Local precipitation of La(OH)_3_ could be detected at these spotted ROIs by the image analysis routine. The onset potential distribution for bare ITO reveals two modes centered at −540 ± 20 mV and −660 ± 50 mV. Although, it seems that the two detected populations can merge together to form a single population, close inspection of the data suggests that there may actually be two or even more populations of average onset potentials related to the structural features of the ITO surface and its surface defects. This high-resolution capability in detecting the different populations seems promising in utilizing this method to study different localized activity-levels on samples due to the presence of different families of particles or surface features.

The same analysis is performed on the MoS_2_-coated ITO electrode in Fig. 4c and e. The detection of surface features depends on the sample illumination, or, equivalently for image post-processing, on the level of image thresholding for automatic ROI detection. The analysis is provided here for two levels of thresholds: a high level to detect a larger number of ROIs (Fig. 4c and d), while a lower level will only highlight the largest objects (Fig. 4e and f).

Fig. 4c shows the onset potential distribution derived from analyzing a large number of ROIs (high threshold) highlighted by red markers in the iSCAT image (Fig. 4d). These ROIs include regions containing MoS_2_ particles as well as areas devoid of particles, such regions/defects of the ITO substrate. Most of these ROIs are actually active towards ORR and, as shown in the graph in Fig. 4c, their activity can be described by two distributions centered at −480 ± 60 mV and −610 ± 30 mV.

The lower threshold employed for image analysis in Fig. 4f, shows three populations of interest. The particles identified for this analysis actually take into account the following two conditions: (i) the particles are detected on the first iSCAT image of the video and/or (ii) the particles are detected in the last image of the background-subtracted video. The population satisfying only condition (ii) corresponds to ROIs which are active towards ORR. The population satisfying conditions (i) and (ii) are classified as MoS_2_ particles active towards ORR and are displayed in Fig. 4f as cyan rings. ROIs satisfying condition (ii) and not (i), displayed as blue rings in Fig. 4f, are ORR-active ROIs but not detected by the low-level threshold (but detected in the high-level one of Fig. 4c and d). Finally, particles satisfying condition (i) and not (ii) are characterized as unactive MoS_2_ particles, displayed as red rings in Fig. 4f. Finally, Fig. 4e summarizes the behavior of the active particles showing the distribution of their respective onset potential (cyan and blue populations respectively). The active MoS_2_ particles (cyan rings) distribution actually overlaps with the main peak centered at −480 mV in Fig. 4c. Compared to the ITO active regions detected in Fig. 4a, the MoS_2_ particles show a decrease by 200 mV of the overpotential for ORR. It confirms the earlier reduction peak detected in the cyclic voltammetry for the MoS_2_ coated electrode (Fig. 2). The higher number of active ROIs detected in Fig. 4c within the −480 mV potential region suggests that the high-level threshold could also highlight MoS_2_ particles smaller than the >350 nm apparent size of the particle detected by the low-level threshold.

The lowest active ROIs centered at −600 mV may be assigned to active ITO defects, which are actually still more active than those spotted in Fig. 4a. It indicates that the electrochemical activity of the ITO substrate can vary between different samples of ITO coverslips. This variability may be attributed to differences in the ITO substrate’s electroactivity, surface composition, and structural defects, which can influence the overall electrochemical reactivity of the substrate. Even if more systematic analysis in an identical manner on several ITO samples would provide higher statistical relevance, this claim confirms the general agreement with other studies regarding the high heterogeneity of ITO electrodes.^51–53^ Moreover, these results can demonstrate the high resolution of detection and high degree of differentiability presented by iSCAT analysis. Fig. 4e allows for example identifying the most active MoS_2_ particles characterized by a *ca.* 200 mV lower overpotential than ITO for ORR.

**Fig. 5:**
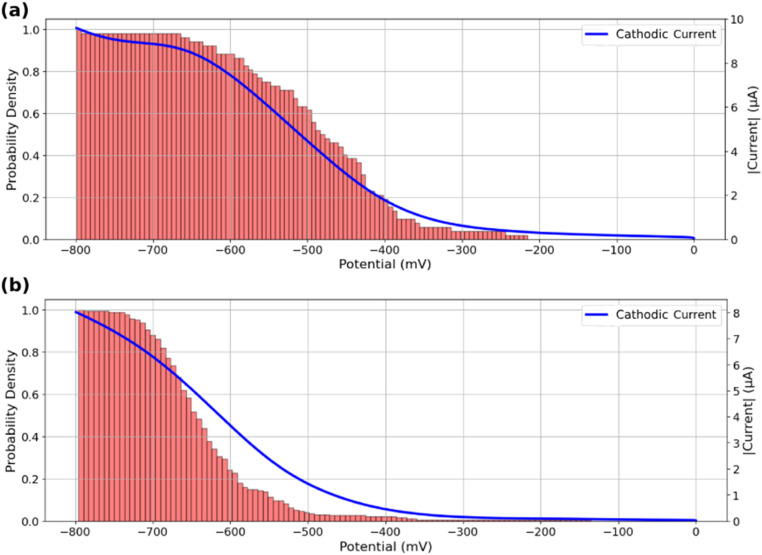
Cumulative histograms of onset potentials, evaluated from the detection threshold corresponding to Fig. 4c, accompanied by the macro-scale electrochemical response of the sample during the potential sweep for (a) MoS_2_ coated ITO electrode and (b) bare ITO electrode.

The analysis of Fig. 4 suggests that active ROIs or features are optically detected in potential regions coinciding with the appearance of faradaic processes recorded on voltammograms, both for bare and MoS_2_-coated ITO electrodes. Fig. 5a and b compare the optical and electrochemical data. They present the cumulative histograms of the onset potential for the MoS_2_ and ITO samples respectively (from Fig. 4c and a respectively), overlaid with their corresponding electrochemical current curves plotted against potential. Particularly Fig. 5a demonstrates a strong correlation between the cathodic current curve obtained from macro-scale electrochemical analysis and the cumulative histograms of onset potentials obtained from localized optical analysis of the MoS_2_-coated electrode using the iSCAT technique. This correlation indicates that as more MoS_2_ particles and ITO active regions contribute to the overall electrochemical reactivity of the sample, the total current increases, reflecting the sum of each individual particle activity. Finally, from the optical identification of the La(OH)_3_ precipitation at individual MoS_2_ particles, one can assign the cathodic peak observed in Fig. 5a to ORR activity at MoS_2_ particles, earlier than the cathodic current observed in Fig. 5b associated to ORR on ITO.

This analysis can also serve as a means of assessing the quality of the region under study in terms of its contribution to the overall reactivity of the sample. Specifically, a greater resemblance between the current curve and the cumulative onset-potential histograms suggests a higher similarity between the behavior of the specific region under study and the behavior of the entire sample at the macro-scale. This correlative study can be used to identify potential inactive regions on a sample or regions exhibiting electrochemical behaviors that differ from the collective behavior of the sample.

The good agreement between the cumulative distribution of onset potentials and electrochemical current during ORR suggests that the particles or defects optically detected in Fig. 4d behave as equivalent objects. It suggests that all particles are either (i) sufficiently spaced to show poor overlap in their diffusion layer for the O_2_ conversion during the ORR, or (ii) are regularly spaced with the same average separation distance (same extent of diffusion layers overlap).^55,56^

**Fig. 6:**
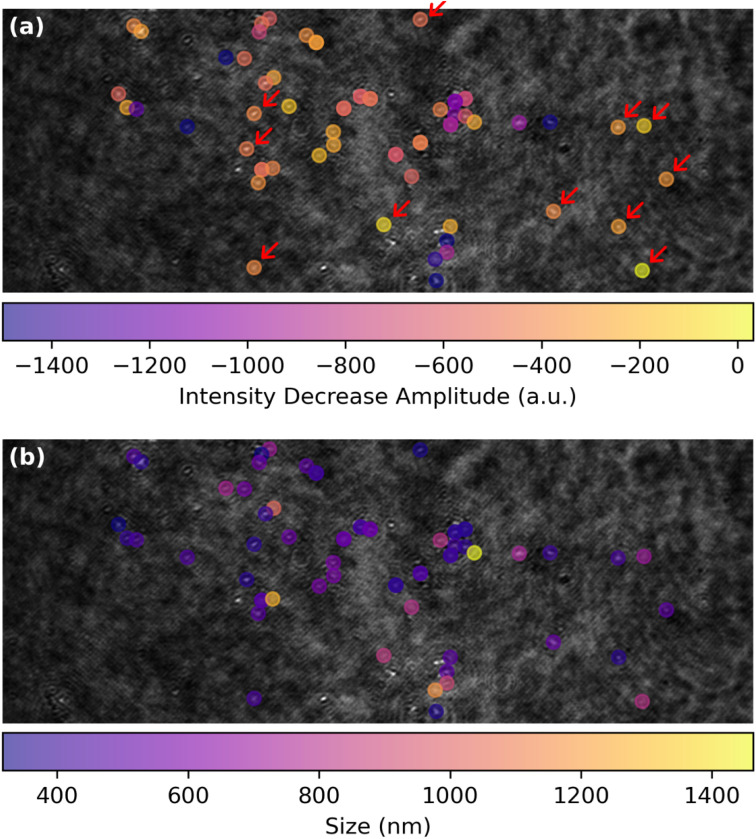
ORR structure–activity maps for single MoS_2_ petal-like particles showing (a) map of the amplitude of the optical intensity decrease, *I*_ORR_ in eqn (3), associated to La(OH)_3_ precipitation during ORR and (b) corresponding map of the apparent optical size of the particles.

The spatial distribution of the MoS_2_ ORR activity can be better apprehended by inspecting in Fig. 6a the maps of the amplitude of the extent of the La(OH)_3_ precipitation, evaluated as the *I*_ORR_ term in eqn (3) obtained from each optical intensity transients sigmoidal fit. This map focuses on the low threshold detection of active particles (Fig. 4f) and shows different close-packed aggregates of particles over the surface along with some, less numerous isolated individual particles (some examples are highlighted by red arrows). Those isolated particles show a roughly −600 a.u. decrease in optical intensity *I*_ORR_ during the ORR.

This map of ORR activity can be compared to the map in Fig. 6b showing the apparent optical size of the active detected particles. Noteworthy, the particles highlighted in Fig. 6a have a comparable size of *ca.* 500 nm. Considering from Fig. 3 that the La(OH)_3_ precipitation should be detected at a level of *I*_ORR_ below −100 a.u., it is suggested that the ORR should be detected by this optical methodology at sub-100 nm nanoparticles.

## Conclusion

This work demonstrates the possibility of using refractive index based optical microscopy to image electrocatalytic reaction at individual particles and at low current density. To access low current density sensitivity, we recourse to the interferometric scattering microscope, iSCAT, which has presented a single biomolecule detection limit, while we focus on the reduction of oxygen, ORR, engaging sub-mM O_2_ concentrations. To be able to probe the ORR, we propose the use of a Lewis acid metallic cation, here La^3+^, which precipitates in the presence of HO^−^. The release of HO^−^ during the ORR can then be probed at the local scale of individual particles from the deposition of a La(OH)_3_ around and atop the catalytic particle. This work presents this methodology by first inspecting the ORR at a transparent and electrocatalytically inert ITO substrate. During the ORR at a bare ITO electrode, some micrometric features are revealed optically, also imaged *ex situ* by SEM, suggesting that the ORR is heterogeneously operated at ITO.

Next the methodology is used to image the extent of the ORR at catalytic MoS_2_ petal-like particles (*ca.* 500 nm in size). From the transient evolution of the La(OH)_3_ deposit around and atop individual particles, the electrocatalytic behavior of individual MoS_2_ particles could be identified through the onset potential of the La(OH)_3_ precipitation and extent of its deposition (assessed optically). Despite the large size distribution of the MoS_2_ particles used herein, the sensitivity of the optical method is discussed suggesting the ORR could be detected at sub-100 nm electrocatalytic nanoparticles.

The method should be generalized to other metallic cations, as other markers for HO^−^ release, for example using the Pourbaix diagram of elements one could generate a Pourbaix-based library of refractive index for optical probing of electrocatalysis at nanoentities. It may also be generalized to the mapping of other acid/base reactions at the nanoscale, and for single particle electrochemistry to other reductive coupled electron–proton transfer reactions.

## Experimental

### Sample preparation

A detailed study on the material structural design is the subject of a future publication. Briefly, 2H-MoS_2_ petal-like nanostructures were prepared hydrothermally using ammonium molybdate tetrahydrate (NH_4_)_6_Mo_7_O_24_·4H_2_O as the molybdenum source and thiourea as the sulfur source over 20 hours at 250 °C. The resulting powder was washed and dried in a vacuum, then dispersed in water and properly diluted until an adequate number of particles were optically observable.

ITO coated coverslips of batch number SPI# 06472-AB with surface area of 22 × 22 mm^2^ and with resistivity of 15–30 Ω manufactured by SPI supplies were used as the transparent conducting substrate. The coverslips were washed 3 times with DI water, ethanol, and acetone and dried under inert atmosphere. MoS_2_ nanoparticles in the powder form were dispersed in DI water and ultra-sonicated for 30 minutes. The concentration of the dispersion was adjusted to be 500 μg ml^−1^. 100 μl of this dispersion was drop-cast on the conductive side of an ITO coverslip and was left in the air atmosphere in an oven in 60 °C overnight. To connect the coverslips to the electrochemical workstation’s wires, a piece of conductive copper tape was attached to the conductive side of the coverslip enabling it to be connected to the workstation using alligator connections. A bare ITO coverslip was used without any further modification as the control sample and to be studied using iSCAT.

### Electrochemical and structural analyses

Electrochemical curves were performed using a CH760E bipotentiostat (IJ Cambria) synchronized with the optical image acquisition. SEM analyses were performed on a Gemini SEM 360 from Zeiss, with an acceleration voltage of 5 kV. The microscope was equipped with an energy dispersive X-ray (EDX) detector from Oxford Instruments. For EDX analysis, the microscope aperture was expanded to 60 μm. Spectra and elemental mappings were processed using the AZtec software.

### iSCAT optical apparatus

The interferometric scattering microscopy (iSCAT) setup is similar to the work described by Ortega Arroyo and Cole.^57,58^ A single mode fiber-coupled 450 nm diode laser (0450L-13A-NI-AT-NF, Matchbox) is collimated to a 10 mm beam with a power of 30 mW. The collimated beam is then focused by a lens (*f* = 250 mm, AC254-250-A-ML, Thorlabs) into the back focal plane of an oil immersion objective (Olympus, 1.40 NA 60×) after passing through a polarizing beam splitter (PBS, PBS251, Thorlabs) and a Quarter-Wave Plate (QWP, WPQ05M-445, Thorlabs). This results in a widefield illumination on the sample. The sample was held by a piezo *Z*-stage which was mounted onto an *XY* stage.

The reflected light at the glass–electrolyte interface and the back-scattered light by the MoS_2_ particles are collected by the objective, and directed back to the QWP and PBS to separate them from the illumination light. On the detection path, the light collected by the objective was sent through a conjugated telescope to reduce the beam by a factor of 6.25 (*f* = 250 mm and *f* = 40 mm, AC254-250-A-ML and AC254-40-A-ML). A final lens (*f* = 100 mm, AC254-100-A-ML, Thorlabs) images the sample onto a CMOS camera (Teledyne Photometrics Prime BSI) with 208× magnification, giving a pixel size of 31.2 nm per pixel.

The focus position is stabilized with an active feedback loop using a total internally-reflected beam. A collimated laser light with wavelength of 633 nm (0633L-11A-NI-NT-NF, Matchbox) was off-axis focused onto the back-focal plane of the objective and the total-internal-reflection occurs at the glass–electrolyte interface. The total-internal-reflected light was collected by the objective and picked up by a micro-mirror, and focused through a cylindrical lens (*f* = 40 mm, LJ1402L2-A, Thorlabs) on to a Quadrant Detector Sensor (PDQ80A, Thorlabs).

### Data acquisition and processing

The raw captured videos were stored as separate HDF5 files containing the intensity values of all the pixels in each frame. The size of each frame was adjusted to be 380 px × 800 px (24 μm × 50 μm) with each pixel representing 62.4 nm (with 2 × 2 pixels binning). The electrochemical data containing potentials, currents, and time-series were then temporally synchronized with the captured video by a National Instruments data acquisition card. A python script was developed to automatically process the data including the optical and electrochemical data. This routine loads all the raw data, adjusts the format of the data to become automatically assessable, stores data as distinct Numpy arrays, and performs further actions to process the data. The frames of the video have undergone averaging processes and a few filtering processes to reduce the background noise and to distinguish useful data from noise-level fluctuations. Minor spatial drifts in the video were corrected using optical flow analysis. The particles in the medium were detected, labeled, and analyzed using the Trackpy library.^59^ The iSCAT frames were constructed by applying moving average filtering on the frames and then subtracting the first frame (averaging from 20 frames) as the background from all later frames.

The kinetics of the ORR process was analyzed using another customized python routine. Inclined sigmoid functions were fitted to the intensity profiles in the vicinity of the detected particles. Based on close inspection of the intensity curves, a comprehensive model was developed to automatically extract the onset potentials of the reactions as accurately as is possible. Finally, the accuracy of this process was inspected carefully and outliers and data points with low quality of data fitting were removed.

## Data availability

Data for this article, including raw iSCAT movies for the bare ITO and MoS_2_-coated ITO in KCl solution in the presence or absence of La^3+^ and respective electrochemical data (voltammograms) are available at Zenodo at https://doi.org/10.5281/zenodo.11624851.

## Conflicts of interest

There are no conflicts to declare.

## Supplementary Material

FD-257-D4FD00132J-s001

FD-257-D4FD00132J-s002

FD-257-D4FD00132J-s003
