# Supplementary material for: Seeing nanoscale electrocatalytic reactions at individual MoS2 particles under an optical microscope: probing sub-mM oxygen reduction reaction
Source: Faraday Discuss. 2024 Jul 10;257:107–25. doi: 10.1039/d4fd00132j (PMC11504976; doi:10.1039/d4fd00132j)
Supplement: FD-257-D4FD00132J-s001 [file FD-257-D4FD00132J-s001.pdf]

Electronic Supplementary Material (ESI) for Faraday Discussions.
This journal is © The Royal Society of Chemistry 2024

Supporting Information

Seeing nanoscale electrocatalytic reactions at individual MoS_2 particles under an optical microscope: probing sub-mM oxygen reduction reaction

Nikan Afsahi,[1] Zhu Zhang,[1,2] Sanli Faez,[2] Jean-Marc Noël,[1] Manas Ranjan Panda,[3,4] Mainak Majumder,[3,4] Naimeh Naseri,[3,4,5] Jean-François Lemineur,[1,*] Frédéric Kanoufi[1,*]

1 Université Paris Cité, CNRS, ITODYS, F-75013 Paris, France

2 Nanophotonics, Debye Institute for Nanomaterials Science, Utrecht University, 3584CC Utrecht, The Netherlands

3 Nanoscale Science and Engineering Laboratory (NSEL), Department of Mechanical and Aerospace Engineering, Monash University, Clayton, VIC, 3800 Australia.

4 ARC Research Hub for Advanced Manufacturing with 2D materials (AM2D), Monash University, Clayton, VIC, 3800 Australia.

5 Department of Physics, Sharif University of Technology, Tehran 11365-9161, Iran.

Email: frederic.kanoufi@u-paris.fr, jean-francois.lemineur@u-paris.fr

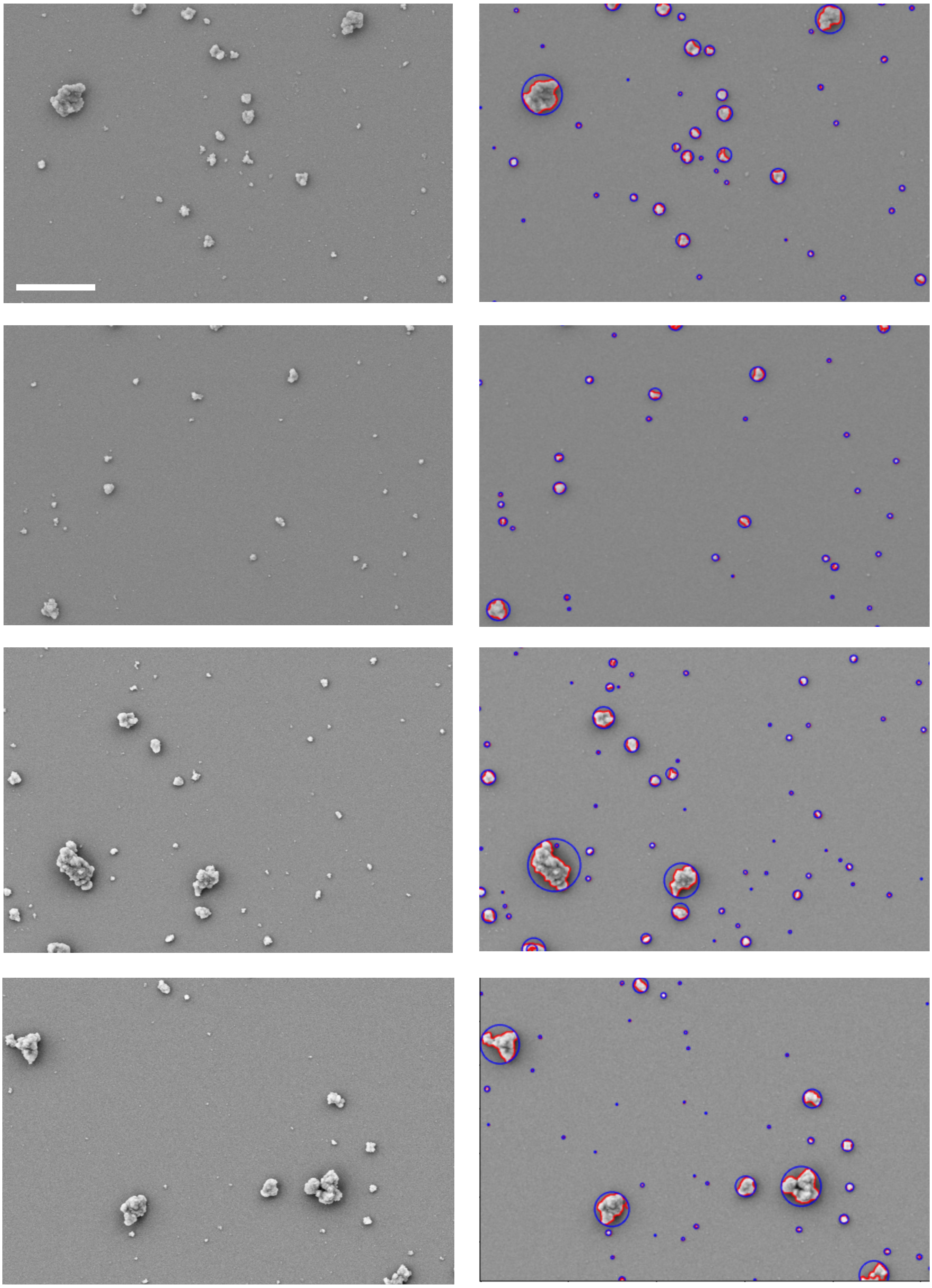

Fig. S1 – Examples of SEM images and the detected particles to calculate the size distribution of the MoS_2 nano- microparticles. (Scale bar: 10 µm, the shown scale bar applies to all the images)

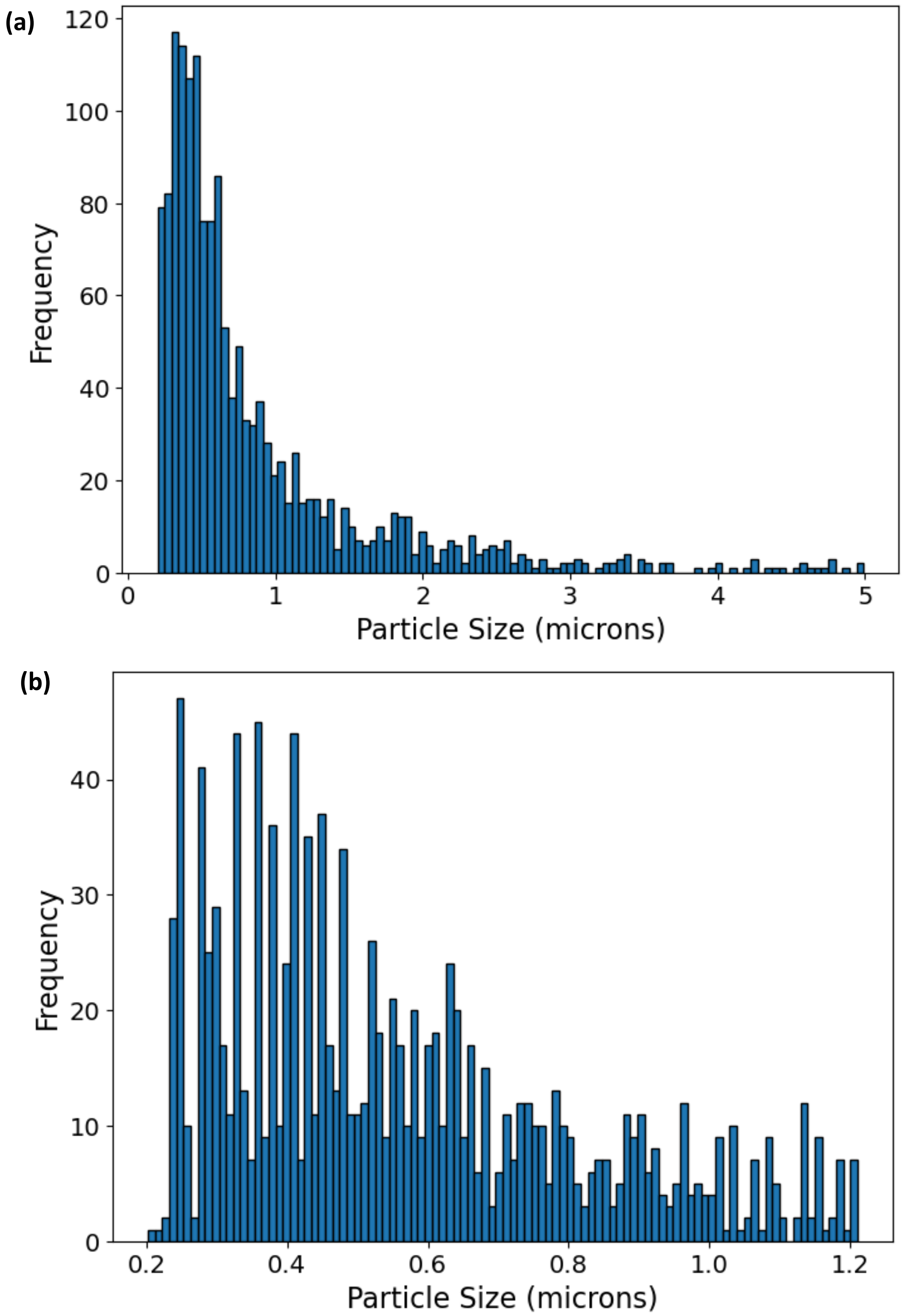

Fig. S2 – (a) Histogram of the size distribution of the MoS_2 particles. (b) Histogram of the 80th percentile of particle size distribution.

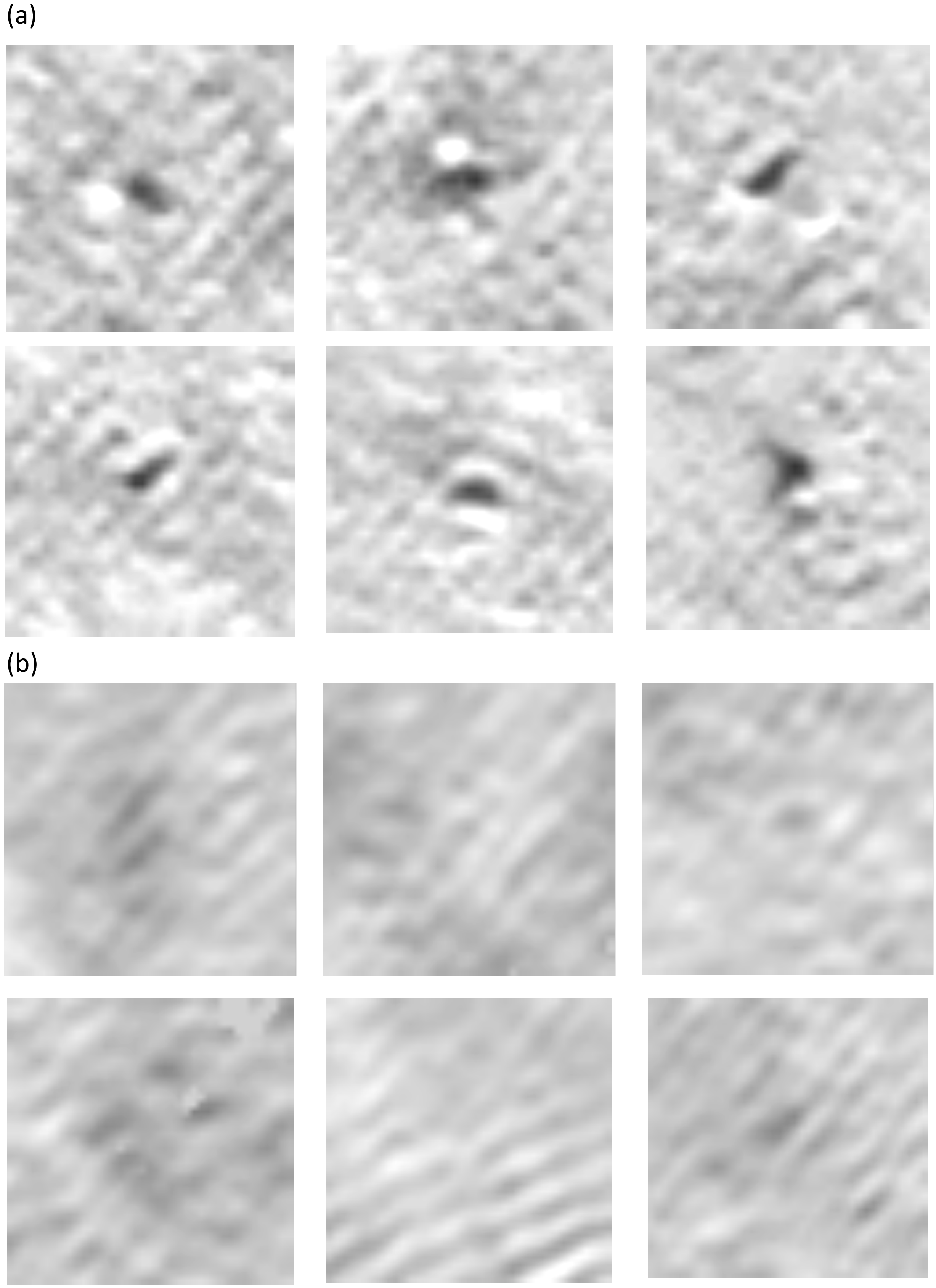

Fig. S3 – Magnified iSCAT images of local regions (2.4 × 2.4 μm^2) after ORR for (a) MoS_2 particles (b) bare ITO.

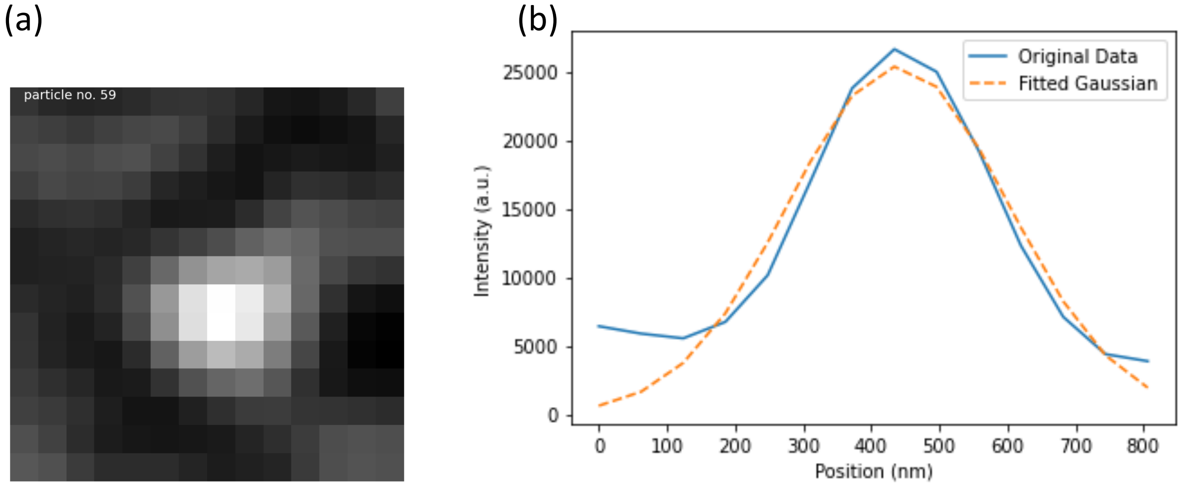

Fig. S4 – Example of (a) a particle detected by optics (the region of interest around the particle is 0.93x0.93 µm^2), and (b) the Gaussian function fitted to its intensity profile. The FWHM of this fitted function represents the particle size.

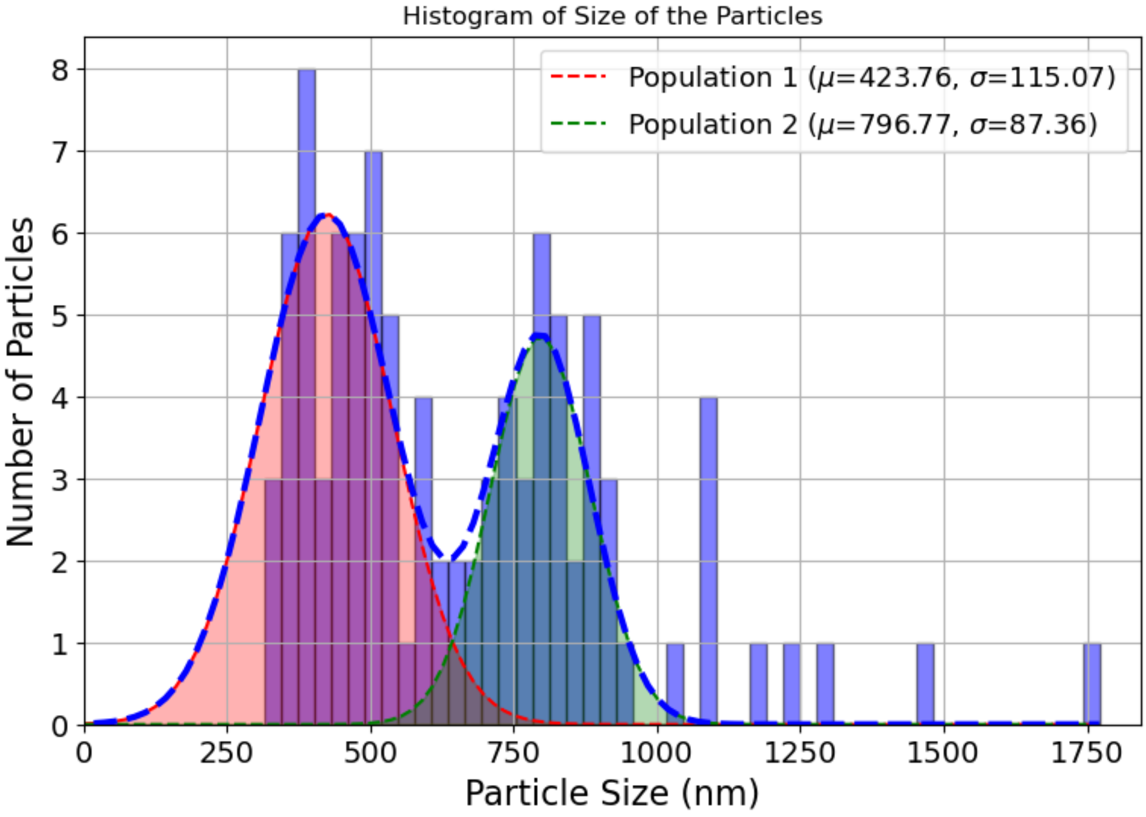

Fig. S5 – The distribution of apparent optical size of particles calculated from the optical image analysis.

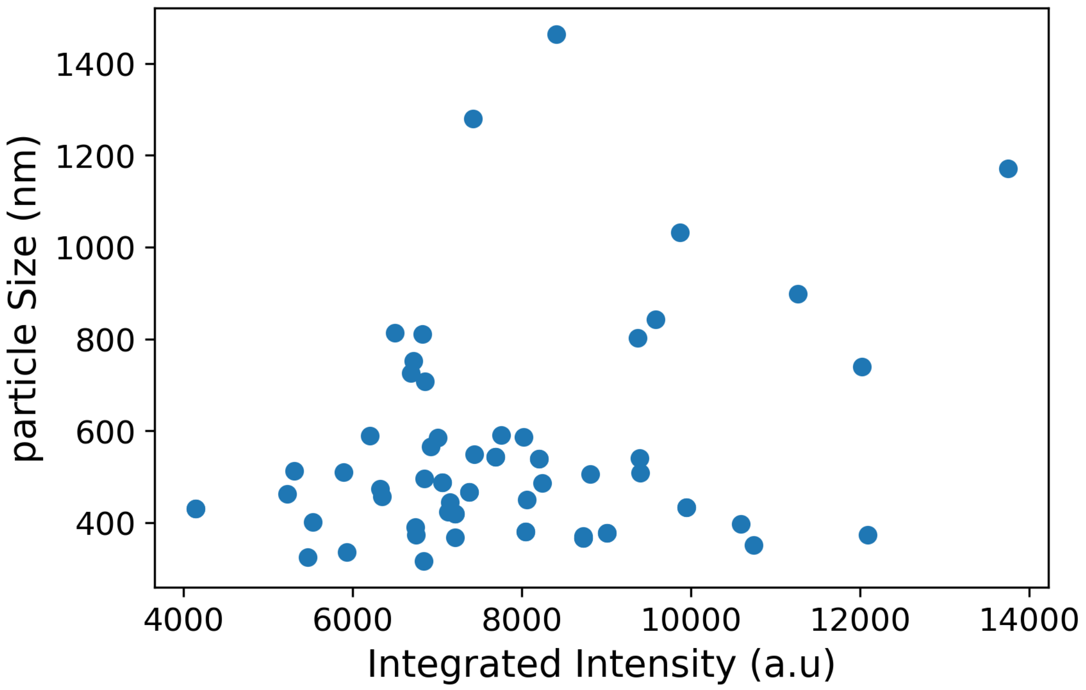

Fig. S6 – Integrated optically intensity (over 15x15 pixels region) of particles vs. apparent optical size of the particles.
